# Editorial: Impact of heavy metal on aquatic life and human health

**DOI:** 10.3389/fgene.2025.1608524

**Published:** 2025-04-23

**Authors:** Ahmed H. El-Sappah, Heba H. Abdel-Kader, Manzar Abbas, Sara Zafar, Jia Li

**Affiliations:** ^1^ College of Agriculture, Forestry, and Food Engineering, Yibin University, Yibin, Sichuan, China; ^2^ Department of Genetics, Faculty of Agriculture, Zagazig University, Zagazig, Egypt; ^3^ Department of Fish Physiology, National Institute of Oceanography and Fisheries (NIOF), Alexandria, Egypt; ^4^ Inner Mongolia Saikexing Institute of Breeding and Reproductive Biotechnology in Domestic Animal, Hohhot, China; ^5^ Botany Department, Government College University, Faisalabad, Punjab, Pakistan

**Keywords:** heavy metal, toxicity, ecosystem, genetic responses, antibiotics

## Heavy metals in aquatic ecosystems: an escalating global concern

Heavy metals’ contamination of aquatic ecosystems is an ever-growing environmental and public health Research Topic. Heavy metals such as cadmium (Cd), arsenic (As), chromium (Cr), lead (Pb), and mercury (Hg) persist in the environment, accumulate in aquatic organisms, and biomagnify through the food chain, ultimately affecting human health (El-Sappah et al., 2017; El-Sappah et al., 2022). Industrialization, agriculture, and urbanization have accelerated the release of these toxic substances into marine and freshwater ecosystems.

This Research Topic seeks to consolidate studies examining heavy metals’ genetic, proteomic, physiological, and environmental effects on aquatic animals while enhancing understanding of their indirect implications for human health. This editorial consolidates the principal results of the papers in this Research Topic and emphasizes their cumulative impact on the field.

## Genomic and proteomic insights into metal stress responses

Understanding organisms’ molecular and genetic responses to heavy metals is crucial for developing biomarkers and mitigation strategies. In this context:

Ebrahim et al. analyzed *Grx4*, *Fep1*, and *Php4* transcription factors in *Schizosaccharomyces pombe*, providing insights into iron homeostasis mechanisms and protein-protein interactions in metal sensing.

Ortega et al. provided a revised glutathione-S-transferase *(GST)* gene map in *Tetrahymena thermophila*, demonstrating stressor-specific gene expression patterns in response to cadmium and arsenic.

These researches underscore the significance of transcriptome and gene regulatory analysis in discerning adaptation mechanisms and possible biomarkers in aquatic creatures subjected to heavy metals.

## Physiological and biochemical effects of metal exposure

In addition to genetic alterations, metal exposure causes significant physiological and metabolic disturbances:

Zhou et al. demonstrated that short-term depuration alleviates cadmium-induced oxidative stress in *Meretrix meretrix*.

Zeng et al. explored the dose-dependent interaction between iron toxicity and the antibiotic norfloxacin in *Larimichthys crocea*.

Ahmed et al. revealed shared stress pathways under salinity and metal exposure in common carp, primarily via immune and hormonal regulation.

These papers demonstrate how heavy metals disrupt physiological homeostasis and provide environmental and aquaculture management insights.

## Environmental monitoring and risk assessment for human health

To safeguard public health, it is essential to evaluate the prevalence and hazards of heavy metals in aquatic ecosystems:

Tolga’s research on Turkish wastewater effluents revealed carcinogenic hazards associated with chromium and nickel.

Zhang et al. demonstrated considerable arsenic deposition in fish from the South China Sea, presenting a concern to consumers.

Wang et al. correlated heavy metal-induced reactive oxygen species production in fish with human sick.

These articles enhance our subject by linking environmental pollution to ecological and human health hazards.

## Expanding omics-based toxicology for environmental health

The use of high-throughput omics technology facilitates comprehensive investigations of metal-induced damage:

Wang et al. used transcriptome and proteomic analyses to investigate phosgene-induced pulmonary damage, emphasizing similarities with processes of metal toxicity.

Collectively, these investigations endorse the ongoing use of integrated omics to comprehend the comprehensive impact of pollutants.

## Conclusion: towards a unified strategy in aquatic toxicology and public health

This Research Topic highlights that heavy metals impact aquatic creatures at several levels, including gene expression, physiology, and ecosystem health, directly affecting human exposure and illness. Future research should prioritize long-term biomonitoring initiatives that include biomarkers across many species and ecosystems. Interdisciplinary methodologies integrating ecology, genetics, environmental chemistry, and public health. Science-informed policy-making to manage discharges and protect ecosystems.

We thank all authors and reviewers for their contributions to this significant compilation. We anticipate it will provide a basis for ongoing research and initiatives to address heavy metal contamination in aquatic ecosystems.
